# Intravenous Fluid Administration and the Coagulation System

**DOI:** 10.3389/fvets.2021.662504

**Published:** 2021-04-15

**Authors:** Corrin J. Boyd, Benjamin M. Brainard, Lisa Smart

**Affiliations:** ^1^School of Veterinary Medicine, College of Science, Health, Engineering and Education, Murdoch University, Murdoch, WA, Australia; ^2^Department of Small Animal Medicine and Surgery, College of Veterinary Medicine, University of Georgia, Athens, GA, United States

**Keywords:** 0.9% saline, colloid, crystalloid, dilution coagulopathy, gelatin, hydroxyethyl starch

## Abstract

Intravenous fluid administration in veterinary patients can alter coagulation function by several mechanisms. Both crystalloid and colloid fluids cause hemodilution, reducing platelet count and plasma coagulation protein concentrations. Hemodilution is associated with a hypercoagulable effect at low dilutions and a hypocoagulable effect at higher dilutions. Composition of crystalloid fluids likely has a minor effect, primarily dependent on fluid ion composition. Hypertonic crystalloids may also cause hypocoagulability. Colloids, both synthetic and natural, can cause hypocoagulability by several mechanisms beyond the effects of hemodilution. These include impaired platelet function, decreased plasma coagulation factor activity, impaired fibrin formation and crosslinking, and accelerated fibrinolysis. The vast majority of the veterinary literature investigates the hypocoagulable effects of hydroxyethyl starch–containing fluids using *in vitro*, experimental, and clinical studies. However, results are inconsistent, likely due to the varying doses and physicochemical properties of the specific fluid products across studies. In addition, some evidence exists for hypocoagulable effects of gelatin and albumin solutions. There is also evidence that these colloids increase the risk of clinical bleeding in people. Limitations of the veterinary evidence for the hypocoagulable effects of colloid fluids include a predominance of *in vitro* studies and *in vivo* studies using healthy subjects, which exclude the interaction of the effects of illness. Therefore, clinical relevance of these effects, especially for low-molecular-weight hydroxyethyl starch, is unknown. Firm recommendations about the most appropriate fluid to use in clinical scenarios cannot be made, although it is prudent to limit the dose of synthetic colloid in at-risk patients. Clinicians should closely monitor relevant coagulation assays and for evidence of hemorrhage in at-risk patients receiving any type of fluid therapy, especially in large volumes.

## Introduction

Intravenous (IV) administration of crystalloid and colloid fluids can affect coagulation function by hemodilution, as well as through factors related to the composition of the fluid (Box 1). The mechanisms are multifactorial, affecting platelet function, coagulation factor concentration, and clot strength, and vary between fluid types. The majority of the literature pertaining to veterinary medicine includes *in vitro* studies, using blood from healthy animals, and *in vivo* studies, administering fluids of concern intravenously to healthy animals. Some studies have also examined effects of fluids on coagulation in experimental animal models of disease, as well as in clinical patients. These study designs each have advantages and limitations. *In vitro* or healthy animal studies are better able to elucidate subtle mechanistic interactions with coagulation, without the confounding effects of illness. However, their clinical relevance may be difficult to interpret. The downside of utilizing disease models or clinical patients is that subtle differences in coagulation are often not detected without large sample sizes because of variability caused by the illness itself or other aspects of clinical management. The authors have endeavored to provide a broad survey of the available data regarding the effects of fluids on coagulation in companion animals. The reader is encouraged to evaluate the breadth of the evidence presented herein and to choose the most relevant data for their specific patients.

Box 1 **Summary of mechanisms by which different fluid types alter coagulation function.**

Crystalloids and colloids

- Hemodilution
  ° Hypercoagulability at low dilutions, possibly due to reduced antithrombin activity
  ° Hypocoagulability at higher dilutions, due to reductions in platelet number and plasma coagulation factor concentration

Colloids (synthetic and natural)

- Impaired platelet function
  ° Reduced expression/accessibility of fibrinogen receptor integrin α_IIb_β_3_
  ° Non-specific binding to the platelet surface
  ° Acquired type 1 von Willebrand disease
- Decreased plasma coagulation factor activity
  ° Specific decrease in factor VIII
  ° Non-specific decrease in all factors
- Impaired fibrin formation and crosslinking
  ° Qualitative differences in fibrin clot
  ° Inhibition of factor XIII interaction with fibrin
  ° Inhibition of thrombin interaction with fibrinogen
- Accelerated fibrinolysis
  ° Enhanced conversion of plasminogen to plasmin
  ° Reduced inhibition of plasmin by α_2_-antiplasmin

## Hemodilution

The dilutional effects of fluid administration on the coagulation system are proportionate to the degree of hemodilution. The effects include decreasing platelet number, but not function, and decreasing procoagulant and anticoagulant [e.g., antithrombin (AT)] proteins to varying degrees (1). The effect of hemodilution is often not the sole purpose of studies assessing coagulation; however, it is often accounted for by the addition of a control group that receives isotonic crystalloid fluid only, or blood diluted with crystalloid only. Therefore, the effects of hemodilution can be surmised by assessing before-and-after changes in control groups within these studies. This evidence on the effects of hemodilution is discussed in detail in the following section on crystalloid fluids.

When comparing the effects on coagulation between fluid types, there are some important considerations concerning hemodilution and the relevant pharmacodynamics of the fluids. It can be challenging to validate the degree of hemodilution between groups in *in vivo* studies. There are a range of methods to measure degree of hemodilution, including utilizing the dilution of endogenous (e.g., hemoglobin, albumin) or exogenous (e.g., indocyanine green) markers, none of which are perfect (2, 3). Thus, the exact degree of hemodilution, or blood volume expansion, caused by different fluid types can vary between studies. However, there is strong evidence that the magnitude and duration of blood volume expansion following administration of synthetic colloid are greater than those of an equal volume of isotonic crystalloid. This may be context-sensitive and dependent on the integrity of the endothelial surface layer, although this relationship is not fully understood (4–7). Studies in healthy dogs (8) and people (9) demonstrated 3- to 4-fold greater hemodilution for dextran, hydroxyethyl starch (HES), and gelatin colloids than isotonic crystalloid. However, the subjects were euvolemic prior to fluid administration, and the fluid doses exceeded common clinical practice. Colloids also had a greater volume effect than crystalloids in healthy dog and pig models of non-traumatic hemorrhagic shock, after fluid redistribution (10–12). Conversely, in an experimental model of orthopedic surgery, dogs administered 10 mL/kg of either high-molecular-weight (MW) HES or balanced isotonic crystalloid showed a similar drop in packed cell volume 1 h postinfusion (13). Clinical trials in critically ill hypovolemic people (14–18) and dogs (19) show that blinded clinicians targeting self-defined clinical endpoints administer on average 1 to 1.5 times the volume of isotonic crystalloid compared to HES, much less than the 3- to 4-fold difference predicted by experimental studies. The targeted endpoints could include normalization of mean arterial blood pressure, heart rate, blood lactate concentration, or central venous pressure, among other clinician-specific criteria. While this may indicate lack of a beneficial colloid osmotic effect in critical illness, it is important to note that the degree of blood volume expansion was not measured in these trials. The volume of fluid administered may simply reflect usual clinical practice. While these uncertainties make it difficult to exactly apportion hypocoagulability following fluid administration into dilutional and non-dilutional components, it is the overall effect that is clinically relevant.

## Crystalloids

The evaluation of *in vitro* crystalloid effects on coagulation has been primarily focused on isotonic crystalloids, although some studies have also evaluated hypertonic solutions. The effect of crystalloids on coagulation is thought to be primarily dilutional; however, variability in the ionic and buffer composition of the fluids may be relevant, specifically with regard to calcium ion content and fluid pH. Most *in vitro* studies have been designed to evaluate the putative effect of a bolus of different amounts of crystalloid, in ratios varying from 4 to 20% of crystalloid to blood, to mimic clinically relevant infusion volumes. Overall, the effects on coagulation appear proportionate to the degree of dilution, with measurable effects generally occurring beyond 20% dilution, or one part fluid to four parts blood.

### Saline 0.9%

Saline 0.9% has been extensively studied because of its use as a diluent for many HES and dextran formulations. In most studies, the dilutional effect on platelet count is not addressed, although it may decrease with increasing dilutions. This may result in some of the recorded changes, especially in platelet dependent assays (e.g., aggregometry). In humans, a 40% *in vitro* dilution of whole blood with 0.9% saline results in decreased platelet function as measured by the Impact cone and plate analyzer (20). Using the platelet function analyzer 100 (PFA-100), *in vitro* 10, 20, 30, and 40% dilutions of human whole blood with 0.9% saline demonstrated a dilution-dependent prolongation of the platelet closure time (PCT) (21, 22). Despite these changes, whole-blood (impedance) platelet aggregometry using adenosine diphosphate (ADP) or collagen as agonists was unaffected by a 5%, 10%, 15%, or 20% *in vitro* dilution with 0.9% saline (22, 23). Platelet aggregometry (impedance or optical) is a low-shear test for platelet function, whereas the PFA-100 uses high-shear conditions. Platelet responses to agonists vary between shear conditions, and platelets under high-shear conditions are reliant on von Willebrand factor (vWF) for adhesion (24). Differences in shear velocities are relevant because they may be representative of platelet performance under venous (low-shear) or arterial (high-shear) conditions. Similar results were seen using optical aggregometry, where aggregation to ADP and collagen was unchanged from baseline at 10% and 20% dilution, but was impaired at 40% (25). It is unclear if platelet count was standardized across dilutions in this study; therefore, absolute platelet count may have affected results. The impairment in this case was not different from that seen with 40% dilution with 6% low MW HES, making it difficult to distinguish between dilutional and functional impairment. In a different assessment of platelet function, the *in vitro* addition of 0.9% saline to human whole blood in dilutions ranging from 2.5 to 30% did not affect platelet P-selectin expression or fibrinogen binding following ADP stimulation (26).

Several *in vitro* studies in dogs have not detected an effect of 0.9% saline on PCT using ADP/collagen cartridges at 10% dilution but have identified mild prolongation at 20% dilution (27–30). One of these studies documented platelet count for each dilution, reflecting that some samples at 20% dilution had a platelet count below the manufacturer's recommended minimum for assessment of platelet function (29). Therefore, the mild prolongation in PCT is likely due to dilution of platelets.

Viscoelastic coagulation evaluation of *in vitro* 0.9% saline dilution of whole blood demonstrated changes consistent with hypercoagulability in some human studies, although it is unclear if this is an artifact or should be a clinical concern. Using thromboelastography (TEG), Roche et al. described slightly increased clot strength and increased rate of clot formation when whole blood was diluted with 0.9% saline at 20% to 40% dilutions (31). At a 60% dilution, the TEG indicated hypocoagulability (31). Other studies have identified decreases in clot strength with a 40% *in vitro* dilution (20, 25). Overall, minimal change in coagulation status occurs at *in vitro* 0.9% saline dilutions <20% (23). Using another viscoelastic measurement (Sonoclot), no changes in clot rate or strength were seen following addition of 0.9% saline to whole blood at 20% or 40% dilutions (32, 33). The slight increase in coagulability with modest hemodilution with 0.9% saline may be due to diluted AT, allowing faster thrombin formation, or may be an artifact. The overall clinical relevance is uncertain. In rotational thromboelastometry (ROTEM) studies of *in vitro* 0.9% saline dilution of whole blood, impaired platelet function appears to play less of a role than fibrinogen in the decreased clot firmness (34–36). This is inferred from greater effects seen on the FibTEM test, where platelet function is excluded, than comparable effects on baseline tests.

In dogs, *in vitro* addition of 0.9% saline to whole blood at a 20% dilution appeared to cause relative hypocoagulability using the ExTEM test, which is a tissue factor–initiated ROTEM test (statistical significance not noted) (27). This was characterized by prolonged time to initiate clotting, decreased speed of clot formation, and lower clot strength. However, another study using 10 and 20% dilutions of canine whole blood failed to document significant changes using ROTEM (37).

*In vivo*, a 10 mL/kg IV infusion of 0.9% saline given to healthy people failed to prolong PCT (collagen/epinephrine and collagen/ADP cartridges), change TEG parameters, or alter flow cytometric assessment of platelet fibrinogen binding or P-selectin expression following activation by ADP or thrombin receptor–activating peptide (21, 38). Another study that administered 1 L of 0.9% saline IV to healthy volunteers demonstrated hypercoagulability on TEG, including shortened R time with increased angle and maximum amplitude (MA) (39). This equates to ~15 mL/kg, resulting in a 9% decrease in hematocrit. This same study documented decreases in fibrinogen and AT concentrations, and the authors hypothesized that the decreased AT may have caused the observed hypercoagulability. A further study by this group, whereby healthy people received 14 mL/kg of a 0.9% saline bolus, showed that the induced hypercoagulable effect was transient, dissipating as the bolus was redistributed (40). Using optical aggregometry, Ruttmann et al. also noted a slightly increased aggregation response to high-dose (100 μmol/L) ADP and to ristocetin (1.2 mg/mL) in humans following a 1 L IV bolus of 0.9% saline (39).

In healthy dogs, a 20 mL/kg IV bolus of 0.9% saline given over an hour resulted in no change in the PCT, which remained within the reference range (41). A hemorrhagic shock study in greyhound dogs compared 80 mL/kg of 0.9% saline to 20 mL/kg of low MW HES administered IV after removal of 48 mL/kg of blood (42). It showed mild prolongation of PCT in both groups, consistent with dilution; however, a slightly greater magnitude of change was observed in dogs that received 0.9% saline. In healthy ponies, an IV infusion of 80 mL/kg of 0.9% saline given over 2 h did not result in changes in cutaneous bleeding time, vWF antigen activity, prothrombin time (PT), or activated partial thromboplastin time (aPTT) (43). Another study of a 10 mL/kg IV bolus of 0.9% saline given to adult horses showed a transient prolongation of PT and aPTT 1 h after the completion of the bolus (44). Additionally, a transient decrease in factor VIII (FVIII) activity was seen in these horses, although there was no change in vWF activity as a result of the bolus (44). Following this bolus, mild transient hypocoagulability was demonstrated using viscoelastic assessment, but platelet function as measured by PFA and optical aggregometry was unaffected (44).

### Polyionic and Ringer Solutions

With regard to hemostasis, the composition of crystalloid (polyionic vs. saline) may result in differential effects, although the magnitude of these effects may not be clinically relevant. One meta-analysis of the human literature suggested that the use of 0.9% saline was associated with greater blood loss compared to lactated Ringer solution (LRS) when used in high-risk patients (45). Proposed mechanisms included the presence of calcium ions in LRS or the effect on coagulation of a saline-induced hyperchloremic acidosis.

Similar to 0.9% saline, the *in vitro* addition of LRS to human whole blood in dilutions ranging from 2.5 to 30% did not affect the expression of P-selectin or fibrinogen binding following ADP stimulation (26). Serial dilutions of human whole blood with LRS did result in significant increases in the rate of clot formation (TEG angle), most prominent at the 30 to 50% dilutions and progressing to hypocoagulability above a 50% dilution (31, 46). In another report, a 33% dilution of human whole blood with LRS did not change the Sonoclot signature from baseline; however, at a 66% dilution, the signature indicated hypocoagulability (32). These changes were corroborated using ROTEM and TEG analysis as well, with significant thrombocytopenia and hypocoagulability following *in vitro* dilution of human whole blood with LRS (47). The hypocoagulable effect of LRS on viscoelastic testing could be ameliorated by correcting the platelet count in the diluted sample (48). Another report evaluating the viscoelastic coagulation monitoring (ROTEM and Sonoclot) effects of *in vitro* dilutions of human whole blood with LRS did not show significant differences (49).

In cats, a 14% dilution of whole blood with Ringer acetate resulted in ROTEM hypocoagulability, as seen by prolongations in clotting time (CT; InTEM assay) and clot formation time (CFT; ExTEM and InTEM assays), and reduced maximum clot firmness (MCF; ExTEM, InTEM, and FibTEM assays) and alpha (ExTEM and InTEM assays) (50). Dose-dependent hypocoagulabilty was demonstrated on TEG analysis with addition of LRS to canine whole blood (16%, 33%, and 66% dilution), showing progressively decreasing angle and MA starting from either 16% dilution or 33% dilution, respectively (51).

*In vivo* studies conducted in healthy individuals have shown mixed results in regard to the effects of administered polyionic solutions; however, most lean toward mild hypocoagulability. A 10 mL/kg IV bolus of LRS given to healthy people failed to prolong PCT using either ADP/collagen or epinephrine/collagen cartridges, compared to baseline (52). In dogs administered a 15 mL/kg IV bolus of modified acetate-containing Ringer solution, no change in PCT or ROTEM values was seen, compared to baseline (53). In the context of experimental severe hemorrhage, dogs resuscitated with LRS (total of 50 mL/kg IV) and stored whole blood (in equal volume to shed blood) showed little alteration in measured PT and aPTT, but appeared to have decreases in activity of factors II, V, VII, VIII, and X, compared to baseline (statistics not shown) (54). In healthy dogs anesthetized for orthopedic surgery that received an IV bolus of 10 mL/kg LRS, PT, aPTT, FVIII:C, and vWF:Ag did not change significantly when measured 1 h after the bolus (13). A mild prolongation of buccal mucosal bleeding time (BMBT) was seen in these dogs, accompanied by a transient decrease in platelet count, but without effects on platelet aggregation in response to platelet activating factor. In rabbits, hemodilution achieved by replacement of 40% of estimated blood volume with five times that volume of LRS resulted in hypocoagulability in both TEG and traditional coagulation testing (55). In this study, it appeared that procoagulant coagulation factors were decreased in excess of anticoagulant factors (e.g., AT, protein C) (55).

Despite the paucity of clinical veterinary studies to draw upon in this area, one observational study in dogs with parvoviral enteritis compared TEG changes in dogs that received bolus fluid therapy (median, 26.5 mL/kg) to those that received a smaller amount (median, 10 mL/kg) (56). Dogs that received bolus therapy showed relative hypercoagulability, but also likely had more severe disease. Resuscitation corresponded with a lower median HCT and higher platelet concentration from admission values for both groups, with a decreased AT activity in the group that received a larger volume of LRS. In both groups, the fibrinogen concentration was above the assay detection limit (700 mg/dL), raising the question of the role of fibrinogen in maintaining *ex vivo* coagulation competency in animals with severe inflammation that are aggressively resuscitated (56).

### Hypertonic Crystalloid

The effects of hypertonic saline on coagulation have been assessed in a limited number of reports. *In vitro* addition of 7.2% saline to canine whole blood prolonged PCT (ADP/collagen cartridge) at both 4% and 10% dilutions, compared to undiluted blood (27). In this study, hypocoagulability was seen in ROTEM analysis, specifically in prolongation of the ExTEM CFT and decrease in MCF. Similar findings were seen when 3% saline was added to canine whole blood at an 11% dilution, with prolonged PCT, increased ExTEM CT and CFT, and reduced ExTEM MCF (57). In dogs with intracranial hypertension given 4 mL/kg of 7.2% saline over 5 min, no significant change in PCT was detected, despite the median PCT being outside of the reference interval immediately after the bolus and 1 h later (58). The only change in ROTEM analysis of these patients was a slightly shortened CT in the FibTEM assay, which is likely not clinically relevant, and was not seen in the prior *in vitro* study (57). In another study where healthy dogs were given 5 mL/kg of 7.5% saline IV at a rate of 1 mL/kg per minute, no change was detected in TEG parameters up to 3 h after the end of the infusion (59).

Overall, the effects of administration of large volumes of crystalloid can cause hypocoagulability, primarily through dilutional effects, and without clear distinction between fluid types. Caution should be used in animals receiving large amounts (>40 mL/kg) of crystalloid, especially in those at increased risk of bleeding. A balanced resuscitation protocol with fresh frozen plasma to maintain concentrations and activity of the key coagulation factors should be considered in patients at increased risk of bleeding (1) with the overall context of the patient kept in mind. Despite these recommendations, the effect of crystalloid on coagulation tests may be transient, considering its redistribution to the extravascular space, and may not necessarily result in clinically relevant hemorrhage, depending on individual patient comorbidities.

## Colloids

Synthetic colloid-containing fluids are commonly administered for fluid resuscitation in small animals (60) and horses (61). They are favored by some veterinarians for their potential to maintain intravascular colloid osmotic pressure and expand blood volume more effectively for a given infused volume than isotonic crystalloids (8, 43, 62). However, they have the potential to cause hypocoagulability through dilutional and non-dilutional mechanisms, including direct effects on platelets and coagulation factors. Along with other adverse effects, the documented clinical relevance of colloid-induced hypocoagulability has led to decreased use of synthetic colloids for fluid resuscitation in human critical care (63). While similar hypocoagulability has been demonstrated in veterinary species, the clinical relevance is unclear at this time. This section will broadly review the mechanistic data for the coagulation effects of synthetic colloids and then detail evidence concerning HES and gelatin separately. Lastly, a brief review of the effects of exogenous albumin administration will be provided.

### Mechanisms of Hypocoagulability

Synthetic colloids cause a dilutional coagulopathy proportionate to the degree of blood volume expansion, which may be of greater magnitude than that from the same infused volume of isotonic crystalloid. There are several additional mechanisms by which synthetic colloids may lead to impairment of coagulation. These include intrinsic platelet dysfunction, acquired von Willebrand disease, a specific decrease in FVIII activity, non-specific decreases in all coagulation factor activity, impaired fibrin formation and crosslinking, and accelerated fibrinolysis.

Reduced cell surface expression, or reduced accessibility for ligand binding, of the activated fibrinogen receptor integrin α_IIb_β_3_ (previously known as glycoprotein IIb–IIIa) has been demonstrated by flow cytometry of human platelets. This includes samples collected from patients undergoing elective surgery treated with HES of varying MW (21, 38) or platelets treated with HES or gelatin *in vitro* (21, 38, 64). Effects of gelatin on this fibrinogen-binding receptor may depend on the type of gelatin tested; reduced, increased, and no effect on expression have all been reported (see below for further discussion on the effect of calcium in diluent) (64). Binding of HES to the platelet surface has also been demonstrated by flow cytometry of human platelets treated with abciximab, a monoclonal antibody that blocks HES binding to integrin α_IIb_β_3_ (65). This is often viewed as non-specific coating of platelets by HES, although the molecular basis is not fully understood. Platelet adhesion may also be impaired because of reduction of the activity of vWF (43, 44, 66–71). A study of human patients administered HES demonstrated an equal decrease in all vWF multimers, characterizing this as acquired type 1 von Willebrand disease (72). Gelatin interferes with collagen binding of vWF, not the absolute concentration; therefore, it may form a complex with the coagulation factor, prompting its excretion (66).

Functional platelet studies have demonstrated a reduction in platelet adhesion and activation, as evidenced by prolonged PCT (21, 38), although not all studies consistently show this effect. Although gelatin interferes with ristocetin-induced aggregation (66, 67, 73–75), it mostly fails to impair aggregation stimulated by other agonists (66, 76–80). The effect of gelatin on PCT is also mixed between species (64, 81).

Given the close association between vWF and FVIII, which circulate as a bound complex, it is unsurprising that synthetic colloids also cause a reduction in the activity of FVIII (67–69, 72, 74, 82, 83). It has been postulated that binding of colloid molecules to the vWF–FVIII complex may accelerate its urinary excretion, although to the authors' knowledge there are no data to support this (72, 84). While the secondary coagulation effects of synthetic colloids are usually described as affecting FVIII specifically, one study of HES administration in dogs showed reductions in other coagulation factors (82). There were concurrent reductions in the concentration of other plasma proteins, along with increases in these proteins in the lymph, leading to the hypothesis that the colloid osmotic effect of synthetic colloids leads to extravascular relocation of proteins. Additionally, an *in vitro* study suggested that AT activity is maintained following hemodilution with HES, in contrast to the decrease in activity seen with crystalloid hemodilution (85).

**Figure 1:**
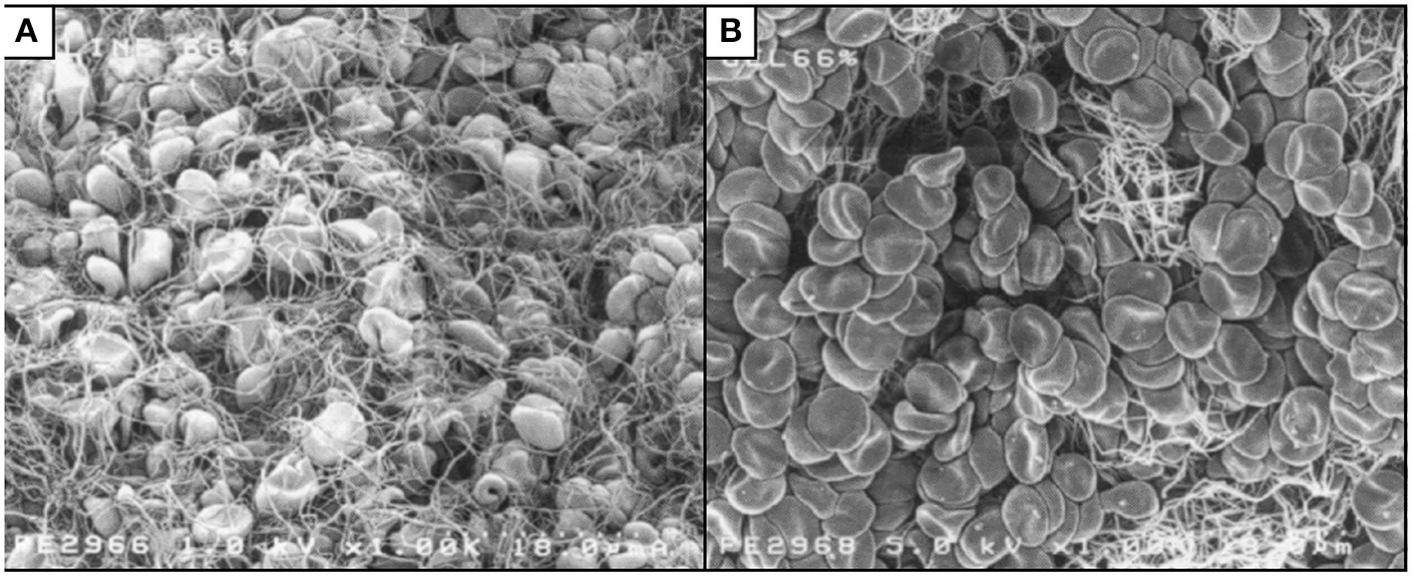
Scanning electron micrographs of whole blood diluted to 66% with 0.9% saline **(A)** or succinylated gelatin **(B)**. Less fibrin meshwork is visible with gelatin dilution. Reproduced with permission (88).

The strength of the fibrin clot is impaired by synthetic colloids. Fibrin clots formed in the presence of HES or gelatin are qualitatively different from those formed in undiluted blood, with increased turbidity and mass/length ratio (86), and reduction in clot density or weight (87–89). Images obtained by scanning electron microscopy show less fibrin meshwork when blood is diluted with either gelatin (Figure 1) or HES, compared to dilution with 0.9% saline (88). Administration of HES to rabbits caused impaired clot propagation and final clot strength by inhibiting the interaction of factor XIII and fibrin, with a secondary mechanism of inhibition of thrombin–fibrinogen interaction (90). Studies in people (*in vitro* and *in vivo*) have also demonstrated a reduction in FXIII, although this is in line with a reduction in other coagulation factors (73, 91). Adding FXIII and fibrinogen back into solution in an *in vitro* study partially corrected coagulation impairment created by gelatin but not that associated with HES (91). Also, infusing fibrinogen concentrate after gelatin in a porcine hemorrhagic shock model improved clot characteristics and reduced blood loss, compared to placebo (89).

Clots formed in the presence of HES are also susceptible to accelerated fibrinolysis. Decreases in the urokinase-activated clot lysis time, a measure of fibrinolysis, have been demonstrated *in vitro* in human plasma mixed with HES and in blood collected from healthy human volunteers administered HES (92). Enhanced conversion of plasminogen to plasmin by urokinase has been demonstrated in the presence of dextran (93). Additionally, dilution of plasma with HES led to reduced inhibition of plasmin by α_2_-antiplasmin (94).

Many of these mechanistic studies are several decades old and were often conducted with a limited range of coagulation assays. For example, whereas some studies have postulated a specific effect against FVIII, most did not measure the activity of other coagulation factors and so cannot exclude broader effects. Additionally, some of the studies have contradictory findings. These may relate to experimental technique, statistical methods, sample size, or differences between the specific colloids investigated. Often the types of colloid studied are no longer in clinical use. Older colloids such as dextran and high MW HES are believed to have greater effects on coagulation than modern low MW HES and gelatin. Thus, the exact mechanisms of colloid-induced hypocoagulability and how much they vary between different types of colloids are still unclear. More recent research, including the bulk of the veterinary literature, has focused on the effects of specific synthetic colloid fluids on clinically applicable coagulation assays and clinical outcomes.

### Hydroxyethyl Starch

The synthetic colloid most commonly administered in veterinary medicine is HES, a synthetic polymer of amylopectin with hydroxyethyl modification at the C2, C3, and C6 positions (60). There are many HES products that differ in concentration, average MW, molar substitution (average number of hydroxyethyl modifications per glucose subunit), C2/C6 ratio (location of hydroxyethylation), the raw material from which they are manufactured (maize or potato), and the carrier solution (balanced crystalloid or 0.9% saline) (84, 95, 96). Higher MW and molar substitution delay degradation and are associated with greater impairment of coagulation. Varieties of HES with MW of >400 kDa are considered high MW, whereas 200–400 kDa is considered medium MW, and <200 kDa is considered low MW. Despite the extensive literature on the effects of HES in many species, this section will focus on the use in dogs, cats, and horses.

*In vitro* studies have mostly demonstrated dose-dependent impairment of coagulation following dilution of blood with different types of HES, compared to crystalloid controls. Common to these studies are measurements of platelet function and viscoelastic tests of coagulation. *In vitro* dilution of canine blood with high MW HES consistently causes hypocoagulability beyond dilution, as measured by TEG (97, 98) and ROTEM (27). Two of those studies also assessed platelet function and did not show any effect beyond dilution on PCT (27, 98). However, in another canine study, dilution with high MW HES showed significant prolongation of PCT, compared to 0.9% saline dilution (30). The only canine *in vitro* study of medium MW HES showed significant prolongation of PCT, compared to 0.9% saline (29). Additional canine *in vitro* studies have used low MW HES, with mixed results. Platelet dysfunction has been demonstrated by changes in PCT (28) or a flow chamber model (99). Other studies measuring PCT have failed to detect an effect beyond dilution with low MW HES (27, 29, 98). Hypocoagulability beyond dilutional effects has also been demonstrated for low MW HES using TEG or ROTEM in three canine studies (27, 37, 98), but was not detected in a fourth (51). The only *in vitro* study of HES dilution of equine blood showed that both high and low MW HES caused significant platelet dysfunction and hypocoagulability, as measured by PCT, TEG, Sonoclot, optical platelet aggregometry, and activities of vWF and FVIII, with many changes in excess of the crystalloid controls (70). The only feline *in vitro* study showed that blood diluted with HES had ROTEM evidence of hypocoagulability beyond that of the balanced isotonic crystalloid control (50). Most *in vitro* studies did not report fibrinolysis data from viscoelastic testing.

A range of *in vivo* experiments have investigated changes in platelet function and viscoelastic testing after HES administration to healthy animals. High MW HES increased PCT in healthy dogs when administered as a 20 mL/kg bolus (41), compared to a crystalloid control, or a constant rate infusion of 1 to 2 mL/kg per hour, compared to baseline (100). Bolus administration of 15 mL/kg of low MW HES to healthy dogs caused PCT prolongation and ROTEM evidence of hypocoagulability beyond dilution (53). However, a slow infusion over 2 h of 10 mL/kg of low MW HES to healthy dogs did not cause any changes in coagulation times or TEG parameters (101). Administration of 10 to 20 mL/kg boluses of high MW HES to healthy horses decreased vWF and FVIII activity (43, 44) and caused PCT prolongation beyond the effects of dilution (44). In the latter study, low MW HES caused similar alterations in coagulation, but of a lower magnitude, and persisting for a shorter time (12 vs. 24 h). Another study of healthy horses that were administered low MW HES showed some evidence of hypocoagulability on TEG with 20 and 40 mL/kg bolus doses (102). However, a different study found no effects on TEG and only minor changes in PT and fibrinogen concentration with a 10 mL/kg bolus (103). The only study of the coagulation effects of low MW HES in healthy foals found that fibrinogen concentration was decreased with a 20 mL/kg bolus, compared to a balanced isotonic crystalloid control (104). Other plasma coagulation assays did not show significant differences to the balanced isotonic crystalloid control, but platelet function and viscoelastic tests were not assessed. As with the *in vitro* experiments, most of these studies did not assess fibrinolysis.

Additional *in vivo* experiments have been performed in models of surgery, hemorrhage, and systemic inflammation. In a canine model of orthopedic surgery, dogs administered a bolus of 10 mL/kg high MW HES were compared to dogs administered the same volume of balanced isotonic crystalloid (13). There were no differences in plasma coagulation assays, platelet aggregation, BMBT, or surgical hemorrhage. Two canine models of hemorrhage showed evidence of hypocoagulability and platelet dysfunction after the administration of 10 to 40 mL/kg boluses of high MW HES, compared to crystalloid, assessed using whole blood and plasma clotting assays and bleeding times (105, 106). Another early canine hemorrhage study showed hypocoagulable TEG parameters after a total of 50 mL/kg high MW HES, but there was no crystalloid control (107). In a subsequent canine hemorrhage model, high or medium MW HES was administered in combination with hypertonic crystalloid at a total dose of 6 mL/kg (108). Only a small prolongation of PT was detected, compared to an autologous whole-blood control. More recently, no difference in PCT was found when 20 mL/kg low MW HES was compared to 80 mL/kg crystalloid for resuscitation in two similar greyhound models of hemorrhagic shock (42, 81). The more recent of these also included ROTEM analysis, with HES administration prolonging the ExTEM CT significantly more than the crystalloid control (81). In that study, the HES group also had evidence of hypocoagulability when compared to a group resuscitated with 20 mL/kg of fresh whole blood. As the crystalloid group had fewer differences compared to fresh whole blood, the study may have been underpowered to detect all of the differences between HES and crystalloid resuscitation. There was no evidence of hyperfibrinolysis. A different canine model of less severe hemorrhage did not find any differences in platelet count, BMBT, PT, aPTT, or ROTEM between groups given low MW HES and crystalloid to cause hemodilution to a hematocrit of 33% (109). A canine model of systemic inflammation showed that administration of 40 mL/kg IV of low MW HES was associated with TEG evidence of hypocoagulability and a decrease in vWF concentration, changes that were greater than the same volume of 0.9% saline (71). That study also had a healthy control group with similar changes after HES administration. Conversely, in a similar equine model, there was no evidence of hypocoagulability when 10 mL/kg of high MW HES was administered in combination with 5 mL/kg of hypertonic crystalloid, compared to 15 or 60 mL/kg of 0.9% saline (110).

There are very few veterinary clinical studies evaluating the effects of HES on coagulation. In a prospective case series, 26 hypoalbuminemic dogs administered variable dosages of high MW HES were monitored for clinical evidence of spontaneous hemorrhage (111). Serial platelet count, PT, and aPTT were measured in 18 of the dogs. Three dogs developed spontaneous hemorrhage, and five had worsening of one or more coagulation parameters. Given the lack of a control group, it is not possible to determine whether these changes were due to HES by a dilutional or non-dilutional mechanism or to the underlying diseases. Historical case series describing the use of high MW HES in combination with buffered isotonic crystalloid to treat hypotension in 21 cats (112) and 16 dogs (113), both to the authors' knowledge only published in abstract form, did not document any excessive bleeding (both dogs and cats) or increases in activated clotting times (cats only). However, there is little detail on how the treatment was administered or how the animals were monitored. A prospective case series of 24 horses with colitis or surgical colic treated with a 10 mL/kg bolus of medium MW HES showed significant prolongation in aPTT postinfusion, compared to baseline, in the subgroup of horses with large intestinal ileus (114). Given the lack of a control group, it is unclear whether this was consistent with hemodilution alone. There was no methodological detail on how horses were monitored for clinical hemorrhage, but there was a statement that no side effects were noted. A more recent prospective case series in 20 hypoalbuminemic dogs administered low MW HES as a constant rate infusion at 1 or 2 mL/kg per hour for 24 h did not detect hypocoagulability with plasma coagulation assays or ROTEM (115). A recent open-label clinical trial randomized 42 dogs with spontaneous hemoperitoneum to initial stabilization with a bolus of either 10 mL/kg low MW HES or 30 mL/kg balanced isotonic crystalloid (116). Immediately after infusion, there was some ROTEM evidence of impaired clot initiation, propagation, and strength in the HES group, compared to the crystalloid group, with no evidence of hyperfibrinolysis. Hemodynamic parameters were not different between groups immediately after infusion, but no subsequent monitoring for hemorrhage was reported. A recent abstract evaluated banked samples from a randomized blinded clinical trial comparing bolus administration of low MW HES or balanced isotonic crystalloid in 39 critically ill dogs (117). No differences between fluid groups were detected in a broad panel of plasma coagulation assays over the 24 h after fluid administration.

While many veterinary studies demonstrate laboratory evidence of hypocoagulability in animals administered HES, data on patient-centered endpoints are lacking. Many human clinical trials have compared blood loss and transfusion requirement between patients administered HES and crystalloid. Meta-analyses of these generally suggest that HES is associated with increased blood loss and transfusion requirement. The latest Cochrane review documented an increased transfusion requirement in critically ill patients treated with HES, compared to crystalloid (118). Another meta-analysis suggested that perioperative crystalloid therapy in patients undergoing major non-cardiovascular surgery resulted in less blood loss, compared to patients who received HES (119). Trauma guidelines recommend avoiding HES because of the adverse effects on hemostasis (120).

### Gelatin

Gelatin-based colloid fluids have been in clinical use since the 1950s (121) and continue to maintain a presence among current fluid choices. There are three different types of gelatin that have been used over the years: oxypolygelatin, modified fluid gelatin (succinylated gelatin), and urea-linked gelatin (polygeline). They all stem from the hydrolysis of collagen but have undergone different chemical processes in order to create a stable fluid product containing molecules of an appropriate size (122). As they have been reported to have various effects on coagulation, it is important to note which type of gelatin is being referred to when assessing any one study. Gelofusine^®^ (4% succinylated gelatin) and Haemacell^®^ (urea-linked gelatin) have dominated the literature in the last 20 years. Brand names are provided in this section when relevant to either currently available products or the diluent of the product.

Most of the evidence concerning gelatin's effect on coagulation stems from *in vitro* and *in vivo* research in people, with few veterinary studies to draw upon. The dominant features of coagulation perturbation include mild platelet dysfunction, likely due to interference with vWF binding to collagen, and interference with fibrin polymerization, causing more friable blood clots. Similar to HES, *in vitro* studies demonstrate a dose-dependent relationship on the effects of gelatin on platelet function and viscoelastic testing, with changes evident beyond ~30% dilution and more consistent at 50% or greater.

Gelatin products inhibit platelet aggregation *in vitro* when ristocetin is used to initiate aggregation. This agonist binds to vWF, which then stimulates the GP1b receptor on platelets. Therefore, it is a test of either decreased vWF or dysfunctional vWF. Impaired response to ristocetin seems to be an effect particular to gelatin. *In vitro* studies using human blood have shown that succinylated gelatin decreases platelet aggregation at >40% dilution, compared to crystalloid dilution (78), although some studies have shown no effect beyond dilutional (77). An *in vitro* study in healthy people found a prolongation in PCT with all three types of gelatin that was similar to dilution with 0.9% saline (64). Inhibition of ristocetin-induced platelet aggregation has also been demonstrated *ex vivo*, when succinylated gelatin was administered to healthy people (66), as well as those undergoing either cardiac or orthopedic surgery (67, 73–75). A canine hemorrhagic shock study found significantly longer PCT after dogs were given 20 mL/kg of succinylated gelatin, compared to the same dose of fresh whole blood or low MW HES, or 80 mL/kg of balanced crystalloid (81).

Compared to similar dilutions with crystalloid, *in vitro* dilutions (10%, 20%, and 40%) of human whole blood with succinylated gelatin cause a greater increase in CFT and greater decrease in alpha angle and MCF on ROTEM, particularly at a 40% dilution (77). Similar concentration-dependent changes have been demonstrated with TEG (123, 124). Some differences in clot rate, time to peak, and MA have also been demonstrated with Sonoclot, with succinylated gelatin having a greater effect compared to crystalloid (32, 33, 125). Studies comparing low or medium MW HES and succinylated gelatin have shown that HES causes a greater magnitude of change in some viscoelastic test parameters, compared to gelatin, although the differences are subtle or inconsistent (49, 124, 126–130).

Some studies have compared different types of gelatin to each other, with the most consistent detrimental effects to coagulation being shown for succinylated gelatin. Haemacell® showed minimal to no effects on Sonoclot testing *in vitro*, whereas succinylated gelatin prolonged clot rate beyond the effects of haemodilution (33). Haemacell® has also been shown to have some hypercoagulable effects on TEG, similar to the addition of 0.9% saline to blood at a low dilution (20%) (131). This mild hypercoagulable effect on TEG parameters was also demonstrated in an earlier euvolemic hemodilution study in dogs (107). Conversely, Haemacell® decreases platelet aggregation in response to a range of agonists, whereas Gelofusine® only causes decreased ristocetin-stimulated aggregation (78). These differences between the fluids can be explained by the presence of calcium within Haemacell®, as the effects of Gelofusine® were similar when calcium was added *in vitro* (78). Oxypolygelatin (Gelifundol®) also appears to cause less effect on viscoelastic measurements compared to succinylated gelatin (Gelafusal®) (123), although oxypolygelatin does inhibit ristocetin-induced platelet aggregation, similar to other gelatins (75). Finally, following *in vitro* activation of platelets obtained from human whole blood diluted with various gelatins, Haemacell® increased the expression of integrin α_IIb_β_3_ receptors, whereas oxypolygelatin (Gelifusin®) had no effect, and succinylated gelatin (Gelifundol®) reduced expression (64).

There are few veterinary reports that document the effects of gelatin on coagulation. Two canine studies assessed platelet function; however, only one controlled for hemodilution (81, 132). This study found a longer PCT after an IV bolus of 20 mL/kg of succinylated gelatin, compared to the same dose of either fresh whole blood or low MW HES, or 80 mL/kg of balanced crystalloid (81). Using a hemorrhagic shock model in dogs, one study showed a slightly increased PT and aPTT after 20 mL/kg of low MW HES given over 20 min, compared to a similar dose of succinylated gelatin (81). Another study in healthy dogs showed no difference in PT or aPTT after 20 mL/kg of succinylated gelatin given IV over 2 h (101). An older study that compared a range of synthetic colloids in a canine euvolemic hemodilution model found effects on PT and aPTT to be comparable between HES and Haemacell® (107). No change in PT and aPTT was found after 5 mL/kg of oxypolygelatin in a second study, compared to baseline (132). Three of the canine studies assessed viscoelastic coagulation (81, 101, 107), with two studies showing more detrimental effects to the speed and strength of clot formation following HES administration, compared to similar doses of either Haemacell® or succinylated gelatin (81, 107).

Clinical outcomes assessed in human patients administered a gelatin product include surgical blood loss, chest drain output, and packed red blood cell transfusions. Chest drain output is a standard measure of blood loss in cardiac surgery. Other outcomes include organ failure scores, hospitalization times, and mortality. Unfortunately, most of these studies have small sample size, impairing their ability to identify definitive differences between fluid types. The two recent large studies with adequate sample size are not randomized trials, which may have introduced bias into the results. Bearing this in mind, one study in cardiac surgical patients found that perioperative administration of succinylated gelatin was associated with a higher chest drain output, higher grades of bleeding, and more patients receiving red blood cell transfusions, compared to the use of crystalloid (133). The second observational report compared separate 2-year periods during which cardiac surgical patients received a majority of succinylated gelatin, low MW HES, or crystalloid in the perioperative period. In this study, more patients received platelet transfusions in the gelatin group than the other groups, and there was a higher risk of in-hospital mortality in the gelatin group (134). Two limitations included the potential bias of time and contamination of the gelatin group with HES prime fluid during cardiopulmonary bypass. Other smaller studies were not able to identify differences in clinical outcomes between fluid types, either gelatin compared to HES or crystalloid (73, 74, 76, 135–140). There are two exceptions concerning studies in cardiac surgical patients. One small randomized clinical trial (*n* = 15 per group) found that patients receiving HES 200/0.5 received more blood products than those receiving succinylated gelatin (137). This finding was similar in another small randomized clinical trial (*n* = 55 per group) comparing HES 200/0.5 to Haemacell® (141). Given the limitations of sample size and observational design, large randomized controlled trials are needed to determine if gelatin fluid products confer increased risk of clinical bleeding. The number of subjects needed to answer this question may not be feasible in veterinary medicine.

### Albumin

The natural colloid albumin also has the potential to cause hypocoagulability. The mechanisms may be similar to synthetic colloids, with impairment of fibrin polymerization a leading hypothesis. Rheologic analysis of samples following *in vitro* dilution of blood from healthy volunteers with 4.5% albumin, low MW HES, or gelatin showed measurements consistent with weaker fibrin clots that were more permeable with less branched polymerization for all three fluids (87). Furthermore, hypocoagulability on ROTEM from *in vitro* albumin dilution can be mostly reversed with the addition of fibrinogen concentrate and factor XIII (142). Impairment of platelet function may also occur, as *in vitro* addition of concentrated albumin to human blood resulted in prolongation of PCT (143). That study also showed ROTEM evidence of hypocoagulability with increasing albumin concentration, despite a method that avoids dilutional effects.

The hypocoagulability caused by albumin appears to be greater than that caused by crystalloids, but less than that caused by synthetic colloids. *In vitro* dilution of human blood with 4% albumin led to greater ROTEM evidence of hypocoagulability than 0.9% saline at a 40% dilution, but not 10 or 20% (144). Another human *in vitro* study showed TEG evidence of hypocoagulability, beyond dilutional effects, with both 30 and 60% dilutions of 5% albumin, but the changes were less than with medium MW HES dilution (124). A similar *in vitro* comparison showed less ROTEM hypocoagulability with albumin dilution than with two low MW HES or succinylated gelatin fluids (128).

Human clinical studies have investigated the effects of 4 or 5% albumin administration on coagulation assays, blood loss, and transfusion requirement. A substudy of the Saline vs. Albumin Fluid Evaluation clinical trial showed that 4% albumin administration was independently associated with aPTT prolongation, but the effect size was small (145). In a clinical trial of cardiac surgery patients randomized to receive LRS, low MW HES, or 5% albumin, there was no difference between groups in the primary outcome of postoperative chest drain output (17). However, both colloid groups received more blood transfusions than the crystalloid group, with no difference between HES and albumin. Both colloids groups showed greater hypocoagulability on ROTEM than crystalloid but with different patterns over time: the HES group was the most hypocoagulable group at ICU admission, whereas the albumin group was most hypocoagulable group at 24 h postoperative. Similarly, in a clinical trial of patients undergoing major surgery, patients randomized to 5% albumin had significantly greater hypocoagulability of TEG parameters than those randomized to LRS, with no difference in blood loss or transfusion (146). There is currently no published veterinary evidence for the effects of either human or canine albumin administration on coagulation. However, as the use of this product potentially increases over time, the effects on coagulation will need to be considered. Additionally, specific indications for albumin in veterinary patients (e.g., hypoalbuminemia due to enteral or renal protein loss) have additional disease-related implications for altered coagulation and should be studied directly.

In summary of the effects of colloids, synthetic colloids and albumin cause a dose-dependent hypocoagulability equal to or greater than that of an equivalent dose of isotonic crystalloids. The evidence for differences between colloid types is not fully elucidated but likely differs based on species and underlying disease. Similar to crystalloids, caution should be used with large doses of colloids and where there are other risk factors for bleeding. Plasma transfusion may partially ameliorate colloid-induced hypocoagulability. However, some adverse effects such as platelet dysfunction may persist.

## Conclusion

The bulk of the available evidence indicates that IV fluids, when administered in clinically relevant doses, can cause dose-dependent hypocoagulability. Synthetic colloids have a greater potential for causing coagulopathy because of multiple non-dilutional mechanisms. The exact differences between fluid products and the clinical relevance of these effects remain an area of investigation. The published body of literature covers a broad range of species and diseases; targeted recommendations for specific populations are not currently possible. Clinicians should be mindful of the potential for hypocoagulability when administering fluids. We recommend closely monitoring relevant coagulation assays (Box 2) and for evidence of hemorrhage in patients receiving fluid therapy and pursuing goal-directed patient-centered resuscitation. Vigilance is especially required in patients with other risk factors for hemorrhage, those receiving a large volume of fluid, or with the use of synthetic colloids. Clinicians should strive for balance across the use of different fluid types and consider replacement of coagulation factors, via plasma transfusion, for bleeding patients where appropriate.

Box 2 **Diagnostic modalities that can detect alterations in coagulation function caused by fluid administration.**

Tests of platelet number and function

- Platelet count
- Bleeding times, such as buccal mucosal bleeding time
- Platelet function analyzer-100 (PFA-100) closure time
- Aggregometry
- Flow cytometry
- von Willebrand factor assays

Tests of plasma coagulation factor function

- Coagulation times (prothrombin time and activated partial thromboplastin time)
- Fibrinogen concentration
- Individual coagulation factor assays

Viscoelastic coagulation tests

- Thromboelastography
- Rotational thromboelastometry
- Sonoclot

## Author Contributions

All authors drafted, revised, and approved final manuscript.

## Conflict of Interest

The authors declare that the research was conducted in the absence of any commercial or financial relationships that could be construed as a potential conflict of interest.

## Abbreviations

ADP: adenosine diphosphate
aPTT: activated partial thromboplastin time
AT: antithrombin
BMBT: buccal mucosal bleeding time
CFT: clot formation time (in the ROTEM assay)
CT: clotting time (in the ROTEM assay)
FVIII: coagulation factor VIII
HES: hydroxyethyl starch
LRS: lactated Ringer solution
MA: maximum amplitude (in the TEG assay)
MCF: maximum clot firmness (in the ROTEM assay)
MW: molecular weight
PCT: platelet closure time (as measured by the PFA-100)
PFA-100: platelet function analyzer
PT: prothrombin time
ROTEM: rotational thromboelastometer
TEG: thromboelastograph
vWF: von Willebrand factor.
